# Comparative analysis of the metal-dependent structural and functional properties of mouse and human SMP30

**DOI:** 10.1371/journal.pone.0218629

**Published:** 2019-06-20

**Authors:** Roshan Kumar Dutta, Fauzia Parween, Md. Summon Hossain, Nidhi Dhama, Parmanand Pandey, Rinkoo Devi Gupta

**Affiliations:** Faculty of Life Sciences and Biotechnology, South Asian University, New Delhi, India; Russian Academy of Medical Sciences, RUSSIAN FEDERATION

## Abstract

Senescence Marker Protein (SMP30) is a metalloenzyme that shows lactonase activity in the ascorbic acid (AA) biosynthesis pathway in non-primate mammals such as a mouse. However, AA biosynthesis does not occur in the primates including humans. Several studies have shown the role of SMP30 in maintaining calcium homeostasis in mammals. In addition, it is also reported to have promiscuous enzyme activity with an organophosphate (OP) substrate. Hence, this study aims to recombinantly express and purify the SMP30 proteins from both mouse and human, and to study their structural alterations and functional deviations in the presence of different divalent metals. For this, mouse SMP30 (MoSMP30) as well as human SMP30 (HuSMP30) were cloned in the bacterial expression vector. Proteins were overexpressed and purified from soluble fractions as well as from inclusion bodies as these proteins were expressed largely in insoluble fractions. The purified proteins were used to study the folding conformations in the presence of different divalent cations (Ca^2+^, Co^2+^, Mg^2+^, and Zn^2+^) with the help of circular dichroism (CD) spectroscopy. It was observed that both MoSMP30 and HuSMP30 acquired native folding conformations. To study the metal-binding affinity, dissociation constant (Kd values) were calculated from UV-VIS titration curve, which showed the highest affinity of MoSMP30 with Zn^2+^. However, HuSMP30 showed the highest affinity with Ca^2+^, suggesting the importance of HuSMP30 in maintaining calcium homeostasis. Enzyme kinetics were performed with γ-Thiobutyrolactone and Demeton-S in the presence of different divalent cations. Interestingly, both the proteins showed lactonase activity in the presence of Ca^2+^. In addition, MoSMP30 and HuSMP30 also showed lactonase activity in the presence of Co^2+^ and Zn^2+^ respectively. Moreover, both the proteins showed OP hydrolase activities in the presence of Ca^2+^ as well as Zn^2+^, suggesting the metal-dependent promiscuous nature of SMP30.

## Introduction

SMP30 is known to possess calcium binding property [1] and is involved in cellular Ca^2+^ homeostasis [2]. Owing to its role in calcium homeostasis, SMP30 plays a major role in various cell processes like cell cycle regulation and apoptosis [3, 4]. Moreover, SMP30 has promiscuous enzyme activity that might vary in the presence of different divalent cations. It is known to show lactonase activity in the ascorbic acid (AA) biosynthesis pathway in non-primate mammals [5, 6]. In addition, it has also been reported to act as organophosphate (OP) hydrolase using diisopropyl phosphorofluoridate as OP substrate [7, 8]. Most of the bacterial OP hydrolyzing enzymes possesses short circulation times *in vivo*, and they have neither the ability to hydrolyze all known toxic OPs nor the high turnover required to dispose off the OPs from blood in one circulation time. However, the bacterial OP hydrolases are likely to initiate potent immune responses in humans; therefore, they are not suitable for repeated use in humans. However, mammalian SMP30, on the other hand, has the potential for providing protection without the complication of inducing an immune response. Hence, recent efforts have focused on identifying potential catalytic bio-scavengers from mammalian sources.

Nerve agents are mostly toxic organophosphates (OP) categorized into two main classes i.e. G-type and V-type. Due to the restricted availability of authentic nerve agents and safety point of view, comparatively less toxic surrogate substrates are used for the laboratory work. Here, in this study, Demeton-S has been used as a surrogate substrate for V-type of nerve agents. Hence, in this study, we aim to clone human (a primate) and mouse (a non-primate) SMP30, express and purify the recombinant proteins of both human and mouse SMP30, and to study the structural conformation and functional deviations in the presence of different divalent metals. Both HuSMP30 and MoSMP30 were cloned in bacterial expression vector. The expressed proteins were recovered from inclusion bodies, and were refolded to make it biologically active. Further, the purified protein was used to study the structural differences with the help of CD spectroscopy. For functional analyses, affinities of the metals were analyzed by UV-VIS titration, and enzyme kinetics were performed in the presence of different metals. These results further supported the metal-dependent enzyme promiscuity shown by SMP30.

## Materials and methods

### Bioinformatics analysis

ExPASy Compute pI/Mw tool was used for estimating the molecular weight and isoelectric point of these proteins. Sequence alignment of human SMP30 (NP_004674.1) and mouse SMP30 (NM_009060.2) was done to compare both the proteins at the amino acid sequence level by using Clustal ω software. ORF finder of NCBI and Translate tool of ExPASy were used for analyzing the reading frame and translation information of genes. Protein structures of MoSMP30 (PDB:4GN7) and HuSMP30 (PDB:3G4E) were taken from the PDB data bank and overlayed using Discovery Studio 4.0 to analyse structural similarity.

### Cloning of SMP30 gene

Mouse SMP30 gene was amplified from kidney cDNA of BALB/c mouse. The kidney tissue was dissected, washed with cold PBS, and minced in liquid nitrogen. Total RNA was isolated using TriZol reagent. PCR was performed to synthesize cDNA using RevertAid H Minus First Strand cDNA Synthesis Kit (Thermo Scientific), following DNase treatment. The synthesized cDNA was used as a template to amplify mouse SMP30 gene using gene specific primers. The purified PCR product was then cloned into pJET vector (Thermo Scientific Clone JET PCR Cloning kit), and verified by colony PCR and restriction digestion (**S1 Fig**). After sequence verification using Sanger DNA sequencing, the gene was sub-cloned into bacterial expression vector pET28a under *EcoR1/Xho1* restriction sites with a 6xHis tag at N-terminus of the gene (**S1 Fig**).

Codon-optimized HuSMP30 gene (NP_004674.1) for expression compatibility in bacterial host system was synthesized from GeneArt, having *NdeI/XhoI* restriction sites for subcloning. The plasmid (from GeneArt) was transformed into *E*. *coli* strain XL-1 blue. Using a single colony from the transformed plate, the plasmid was isolated and digested with *NdeI/XhoI* restriction enzymes (**S2 Fig**). The insert of gene HuSMP30 was prepared by gel elution using MinElute gel extraction kit (Qiagen). Similarly, vector pET28a was prepared by digesting with the same restriction enzymes (**S2 Fig**). The ligation was performed using T4 DNA ligase, and the ligation mixture was transformed into *E*. *coli* (XL-1 blue). The positive clones were analyzed by colony PCR and restriction digestion. Subsequent confirmation of the positive clone was done by Sanger DNA sequencing using primer for T7 promoter.

### Expression of recombinant human and mouse SMP30 proteins

Both MoSMP30 and HuSMP30 genes were cloned in pET28a vector, and transformed in *E*. *coli* (BL21, DE3) cells. The transformed cells were grown overnight on LB agar plates in the presence of kanamycin (50μg/ml) at 37°C temperature. A single colony was picked for primary culture in 5 ml of LB broth with kanamycin and grown at 37°C overnight with 260 rpm in a shaker incubator. Subsequently, secondary culture (500 ml of LB broth with Kanamycin) was set by inoculating primary culture in 1:100 dilution, and grown for 2–3 hours till OD_600_ ~0.5. The secondary culture was induced with 0.1mM IPTG, and then it was grown at 23°C for overnight in the shaker incubator with 260 rpm. Then the cells were harvested using Sorvall RC 6 Plus centrifuge (Thermo Scientific, USA) at 4,000 rpm for 20 minutes at 4°C. The cell pellets were re-suspended in sonication buffer (50 mM Tris-HCl pH 8.0, with 0.5% Triton X-100, lysozyme 1.0 mg/ml, and 1 mM PMSF). The resuspended cells were then sonicated using sonicator (SONICS, USA) at 4°C for 5–6 cycles of 20 seconds pulse, and 20 seconds break at 30% amplitude. The cell lysates were centrifuged at 8,000 rpm at 4°C for 20 minutes. The crude supernatants were collected, and the pellets were re-suspended with the same volume of sonication buffer without lysozyme. Finally, the expression level in all respective supernatants and pellets were analyzed by 12% SDS-PAGE (**S3 Fig**).

### Purification of soluble proteins by affinity and size exclusion chromatography

The supernatants obtained after centrifugation of crude lysate were purified by Ni-NTA column using FPLC. The column was first washed with lysis buffer (50 mM Tris-HCl pH 8.0, with 0.5% Triton X-100 and 1 mM PMSF) using ten times volume of the column, followed by washing with 50 mM imidazole containing lysis buffer to remove any non-specific bound proteins. The bound proteins were then eluted in five different 0.5 ml fractions with elution buffer (50 mM Tris-HCl pH 8.0, with 0.5% Triton X-100, 1 mM PMSF, 200 mM imidazole). All fractions including flow through and wash were analyzed on 12% SDS-PAGE (**S4 Fig**).

The purified MoSMP30 and HuSMP30 proteins from Ni-NTA column were further purified by size exclusion chromatography using Superose 12 (10/300) column. PBS (50 mM phosphate buffer pH 7.4 and 150 mM NaCl) buffer was used for both column equilibration and sample application. All the peaks greater than 10 mAU were collected separately by using FPLC fraction collector, and further concentrated using Millipore-Amicone filter 0.5 ml Millipore-Amicone 15 ml filter. Purified proteins were stored in 50 mM phosphate buffer (pH 7.4) with 150 mM NaCl at -20°C, and utilized within a week.

### Isolation of SMP30 through inclusion body solubilization

Proteins from inclusion bodies were purified using a previously published method with some modifications[9,10]. Briefly, cells were harvested from the 500 ml of secondary culture by centrifugation at 7,000 rpm for 20 min at 4°C. Pelleted cells were resuspended in 70 ml buffer containing 50 mM Tris-HCl pH 8.0, and 1mM PMSF. After resuspension, 4.0 ml lysozyme (10 mg/ml) was added into the resuspended cells and kept at room temperature for 1 hour on a rocker for proper mixing. Subsequently, 12.5 ml of 5M NaCl was added into it, and mixed well. Then 10 ml of 25% Titron X-100 was added, mixed well, and then kept at room temperature on a rocker for mixing. The solutions were sonicated at 40% amplitude 1-seconds on/off conditions for 1 minute, and this step was repeated for six times. The lysed cells were centrifuged at 11,000 rpm for 20 minutes at 4°C. The supernatants were stored, and the pellet was resuspended in 48 ml buffer (50 mM Tris-HCl pH 8.0, 1 mM PMSF) and 2 ml of 25% Titron X-100. This step of resuspension, sonication and centrifugation were repeated for five times using the same buffer without Triton X-100. Finally, 20 ml protein extraction buffer {3M Guanidin hydrochloride (GdnHCl), 50 mM Tris-HCl pH 8.0, and 1 mM PMSF} was added to the pellets for dissolving the inclusion body proteins, and incubated at room temprature on a rocker for 10 minutes for completely dissolving the proteins. It was then centrifuged at 20,000 rpm for 20 minutes at 4°C, the supernatants were collected, and concentrations of the proteins were measured using NanoDrop (ThermoScientific, USA). The solubilized proteins were dialysed twice for the slow removal of GdnHCl using dialysis buffer containing 2 M GdnHCl followed by 1 M of GdnHCl. All the fractions collected were analysed on 12% SDS-PAGE (**S5 Fig**). Both of the proteins were alliquoted into four 15 ml conical centrifuge tubes, and divalent metals (CaCl_2_, CoCl_2_, MgCl_2,_ and ZnCl_2_; final concentration 1 mM) were added in each of the 15 ml conical centrifuge tubes separately and kept on a rocker for 5 minutes at room temprature for proper folding. Finally, the protein concentration were estimated using BCA reagent (Pierce BCA Protein assay kit, Thermo Fischer) and BSA as a standard.

#### Circular dichroism (CD) spectroscopy

Purified proteins were used at concentration of 0.5 mg/ml with metal co-factors CaCl_2_, CoCl_2_, MgCl_2_, and ZnCl_2_ for the structural analysis using CD spectroscopy (Applied photophysics Chirascan with stop flow instrument, Surrey, UK). The samples were prepared in buffer (5 mM Tris-HCl pH 8.0, 0.2 μM PMSF and 5 mM GdnHCl) with different concentrations of divalent metals (2 mM, 5 mM, and 10 mM) keeping total volume 400 μl. Each of the samples were scanned thrice, and the spectrum was recorded between 190 nm to 260 nm wavelenth. CD spectra of buffer alone were used as blank, and substracted from the experimental data. CDNN software was used to analyse the data.

#### UV-VIS analysis for the calculation of dissociation constant (Kd)

Kd values were calculated using UV-VIS titration following the method described earlier [11]. Both MoSMP30 and HuSMP30 proteins were purified from inclusion bodies, dialysed, and the affinities were meassured with diferent divalent cation (Ca^2+^, Co^2+^, Mg^2+^, and Zn^2+^). For the titration, protein samples (73.53 ηM) were prepared in buffer (50 mM Tris-HCl pH 8.0, 0.15% Titron X-100) with varied metal concentrations, i.e. from 1 μm to 1 mM. Background level of absorbance was meassured by adding 1mM EDTA to chelate all the metals from the protein sample. The prepared samples were incubated for 20 minutes and then UV-VIS spectrophotometer (Lambda 45 from Perkin Elmer) was used to perform the scanning of the protein metal complex ranging from 250 nm to 500 nm wavelength. Then, delta absorbance were calculated by substracting the background level of absorbance. A concentration dependent shift in the absorbance was observed at 333nm (**S6 Fig**). The average values of delta absorbance at 333nm were plotted using Origin pro software for MoSMP30 (**S7 Fig**) and HuSMP30 (**S8 Fig**). The Kd values were calculated by curve fit nonlinear fitting of the data using the following Hill equation-

Y=Vmax*X^n/(K^n+X^n)

where Y is delta absorbance at 333nm, Vmax is maximum delta absorbance, X is the metal concentration, K is the metal concentration at which reaction rises, and n is the Hill co-efficient which provides a measure of the cooperativity of substrate binding to the enzyme[12].

### Enzyme kinetics with γ-Thiobutyrolactone and Demeton-S

Enzyme kinetics were performed following the modified methods reported by Kondo et al. [7]. Proteins purified from inclusion bodies were used to perform lactonase and OP hydrolase activities, for which, γ-Thiobutyrolactone (GTBL) and Demeton-S (Sigma Aldrich) were used as substrates to measure lactonase and OP hydrolase activities respectively. Divalent metals CaCl_2_, CoCl_2_, MgCl_2_, and ZnCl_2_ were used as cofactors to study the metal dependent activities of SMP30. Enzyme kinetics was performed in activity buffer containing 50 mM Tris-HCl pH 8.0; 1 mM divalent metals for OP hydrolysis activity and activity buffer containing 50 mM Tris-HCl pH 7.0, 1 mM divalent metals for lactonase activity. Different substrate concentrations (1 mM, 2.5 mM, 5 mM, and 10 mM) of each were used in the reactions, and the concentration of proteins used in the reactions was 73.53 ηM. All the reactions were performed in triplicate in 96 well plates keeping reaction volume 200 μl. For both the activities, 0.1 mM 5,5’-Dithiobis, 2-nitrobenzoic acid (DTNB) was used as an indicator. The substrates and indicator were prepared in activity buffer by keeping the volume 100 μl for each reaction. Enzymes were also prepared in activity buffer separately in 100 μl, and were mixed with substrate just before reading starts. The kinetics was performed at 405 nm upto 30 minutes with the intervals of 1 minute using multimode micro-plate Reader (BioTek Synergy HT). The product formation (OD/min) was plotted using double reciprocal graph (Lineweaver Burk plot) and Vmax & Km values were estimated. The graph was plotted taking the average values of each triplicate with the standard error of the mean (SEM) in the plot. Further, turnover number ‘Kcat’ was calculated taking epsilon value (14103.33 M^-1^ cm^-1^) in consideration of DTNB (D8130-10G, SIGMA), and dividing Vmax value with final protein concentration (73.53 ηM) used in the reaction.

## Results

### Comparative bioinformatics analysis

**Fig 1:**
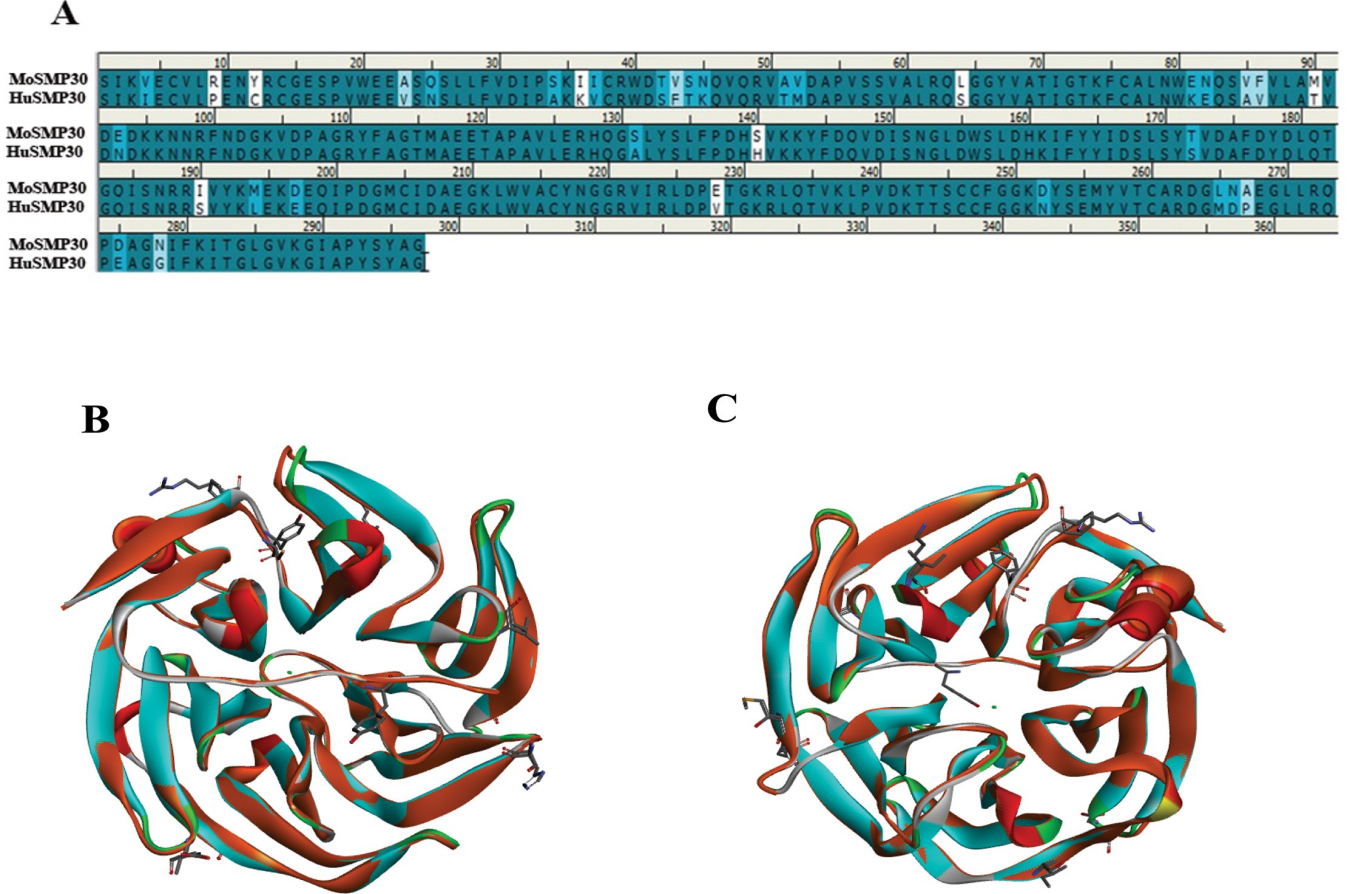
*In silico analysis of MoSMP30 and HuSMP30*. (**A)** Sequence alignment of MoSMP30 and HuSMP30 protein shows 88.6% sequence identity and 95.3% sequence similarity. (**B**) Superimposition of MoSMP30 (Cyan) and HuSMP30 (Orange) shows minor deviation in C^α^ level (RMSD = 0.52Å). The dissimilar amino acid residues are presented by stick model. Cyan dot in the center represents the metal, as SMP30 is a metalloprotein (**C**) The top view of the superimposed crystal structure of Mouse and Human SMP30. The crystal structures were retrieved from the RCSB PDB database, and the accession ID for MoSMP30 and HuSMP30 are 4GN7 and 3G4E respectively. The analysis was performed in Discovery Studio 4.0.

The sequence alignment of HuSMP30 (NP_004674.1) and MoSMP30 (NM_009060.2) showed 88.6% sequence identity and 95.3% similarity at amino acid level (**Fig 1**). Protein structures of MoSMP30 (PDB:4GN7) and HuSMP30 (PDB:3G4E) were overlayed using Discovery Studio 4.0 to analyse structural similarity. Superimposition of both the protein structures showed a minor deviation (RMSD = 0.52Å) in the C^α^ atoms level of the proteins. There were only eight highly dissimilar amino acid residues mainly present in the outer shell, and not in the active site of the SMP30 (**Fig 1**), suggesting an evolutionarily conserved proteins in the mammals.

### Recombinant expression and purification

**Fig 2:**
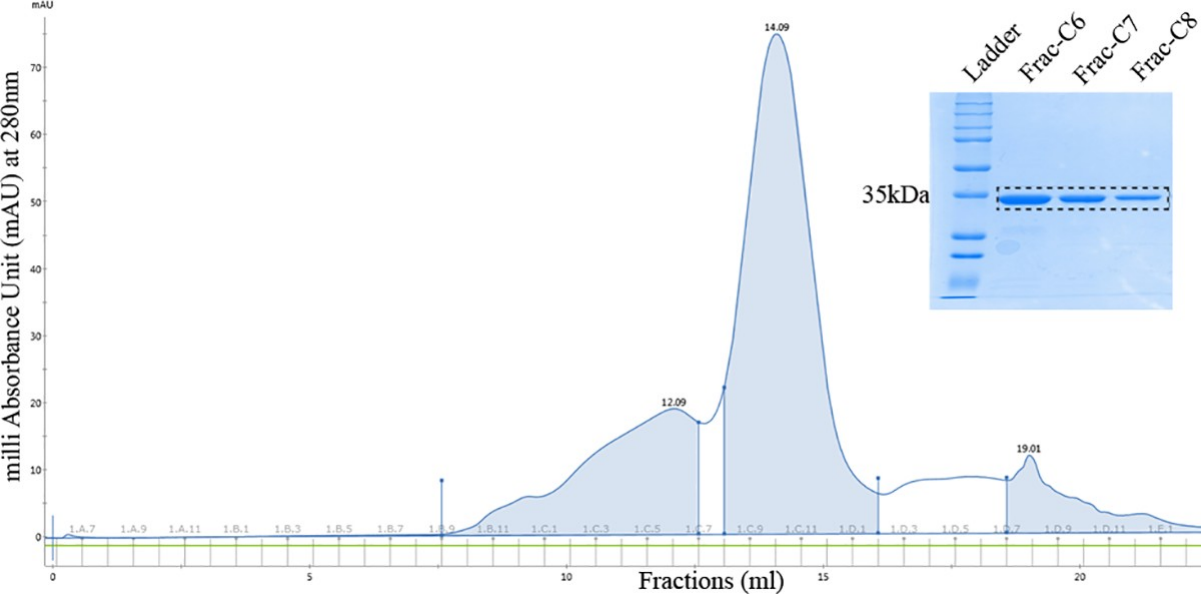
Purification of HuSMP30 by gel filtration chromatography. The elution chromatogram and eluted fractions (C6, C7, C8) from the column superose 12 10/300GL.

**Fig 3:**
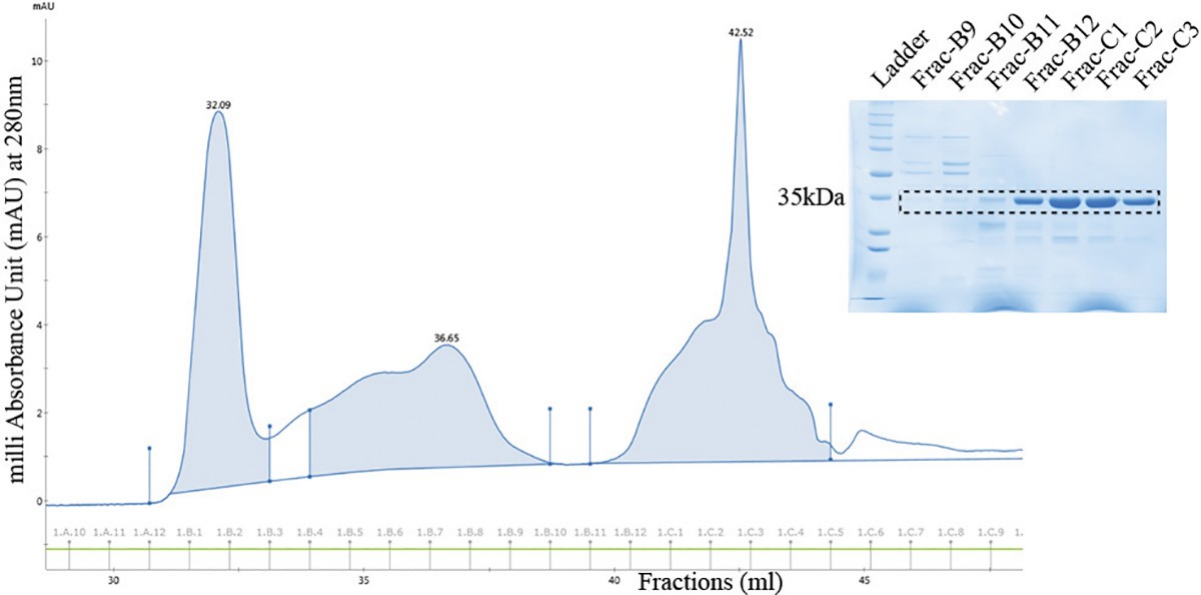
Purification of MoSMP30 by gel filtration chromatography. The elution chromatogram and fractions collected (B9, B10, B11, B12, C1, C2, and C3) from the column superose 12 10/300GL.

The MoSMP30 gene amplified from the cDNA prepared using mouse kidney and codon optimized HuSMP30 gene were cloned in pET28a with 6xHis tag at N-terminus (**S1 and S2 Figs**). After sequence verification, these plasmids were transformed in *E*. *coli* (BL21) cells for protein expressions. SDS-PAGE analysis showed that both MoSMP30 and HuSMP30 proteins were significantly expressed after IPTG induction. However, ~90% of the total recombinant proteins were in the insoluble pellet fractions, and only ~10% of the proteins were in supernatants (**S3 Fig**). Initially, the supernatants were used to purify proteins by Ni-NTA affinity chromatography which showed little impurity (**S4 Fig**). Hence, Ni-NTA purified HuSMP30, and MoSMP30 proteins were further purified by size exclusion chromatography (**Figs 2 and 3**). The cell pellets obtained after cell lysis were utilized to purify inclusion bodies as most of the proteins were in insoluble fractions. The inclusion bodies were purified, and solubilized in the extraction buffer (**S5 Fig**). Total yield of the proteins from inclusion bodies were calculated by estimating protein concentrations, which were found to be 272.6 mg/L culture for MoSMP30 and 133.4 mg/L culture for HuSMP30.

### Biophysical characterization of SMP 30

**Fig 4:**
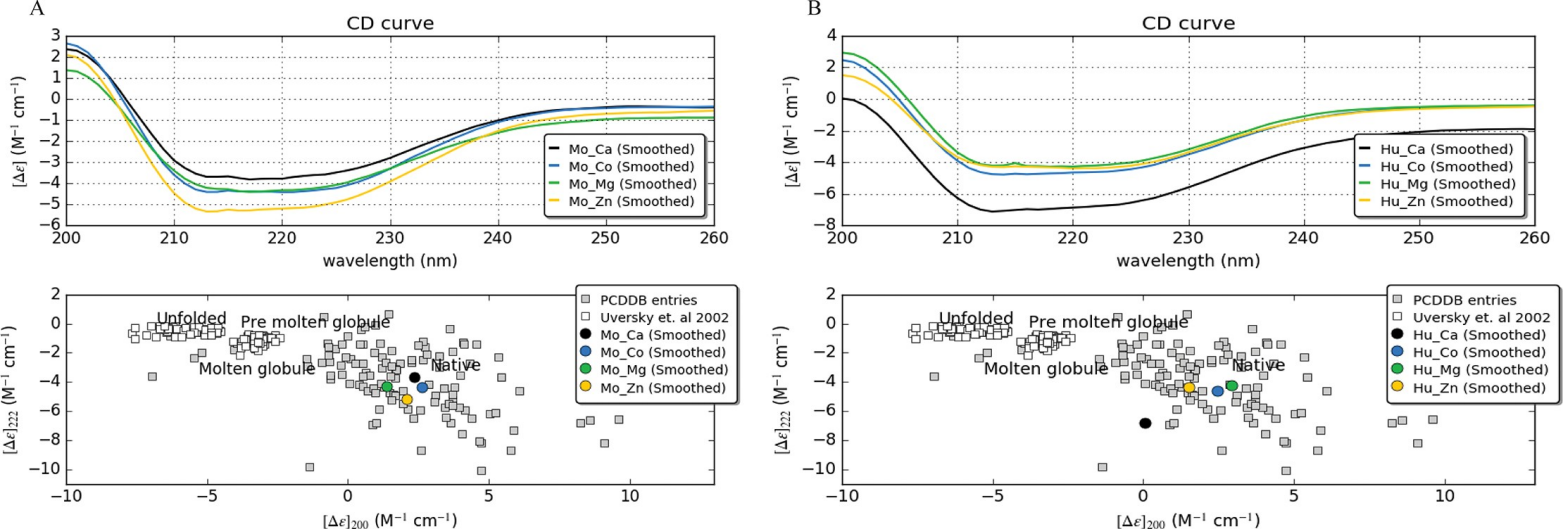
CD spectra of the MoSMP30 and HuSMP30. CD spectrum ranges 200 nm to 260 nm was used and scanned the solution three times at room temperature. The representation of the spectrum is the average of the three scanned ODs which was reduced from the blank (buffer without protein). The spectrum is represented for **A**). MoSMP30 and, **B**) HuSMP30 with 10 mM of divalent cations Ca^2+^, Co^2+^, Mg^2+^ and Zn^2+^. Δε represents delta epsilon, a molar circular dichroism plotted with CAPITO software. Lower panel shows the folding states of both **A**) MoSMP30 and **B**) HuSMP30 proteins in the presence of different metals. CD values at λ = 200 nm versus values at λ = 222 nm were plotted to deduce the folding states.

**Fig 5:**
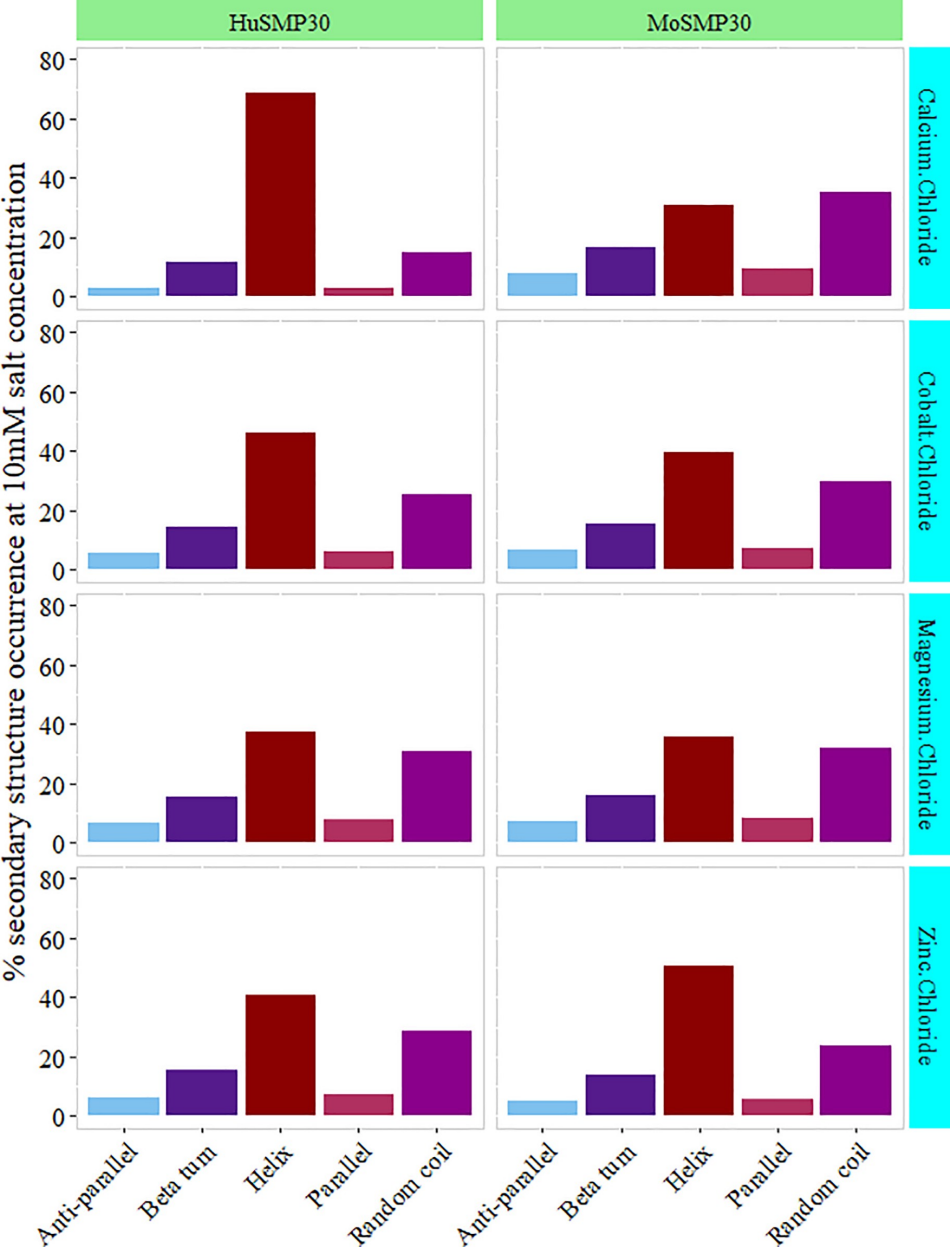
Variations in secondary structures. Different divalent cations (i.e. Ca^2+^, Co^2+^, Mg^2+^ and Zn^2+^) with 10 mM concentration were used to generate the primary CD spectral data. Further, this data was processed in CDNN software to predict percent protein secondary structure conformation in a different state. CD spectra from 200 nm to 260 nm ranges were plotted in the graph. The plotted values are the result of blank (buffer without protein) subtraction and an average of triplicate samples.

Primary CD data was measured within 190–260 nm spectral windows. The presence of different metal contents (CaCl_2_, CoCl_2_, MgCl_2_, and ZnCl_2_) and the varied concentrations (2 mM, 5 mM, and 10 mM) resulted in native conformations of both the proteins (**Fig 4**). The CD spectral data was further analyzed by CDNN program, which resulted in a prediction based secondary structure observation [13, 14]. The percentile values of different secondary structure conformations of MoSMP30 and HuSMP30 were obtained as α-helices (Helix), β-sheets (anti parallel, parallel, and beta turn), and random coils [15]. Slight structural dissimilarities in the secondary structures, especially in the helix conformation, were observed in the presence of different metals **(Fig 5)**. Interestingly, concentration dependent attainments of native conformations were observed in the presence of Zn^2+^ for MoSMP30 **(S1 Table)** and Ca^2+^ for HuSMP30 **(S2 Table)**.

To study the comparative metal affinity, Kd values were calculated using UV-VIS spectra **(S6 Fig)**. Both the proteins showed affinity towards metal cations, however, the affinity patterns were found to be different (**Table 1**). MoSMP30 showed highest affinity with Zn^2+^ followed by Mg^2+^, Ca^2+^ and then Co^2+^ (**S7 Fig**). With HuSMP30, the lowest Kd value was observed with Ca^2+^, confirming the highest affinity with Ca^2+^ followed by Zn^2+^, Mg^2+^, and Co^2^ (**S8 Fig**).

**Table 1:** The K_d_ values of both the proteins with different metals.

Metals	MoSMP30 / Kd (μM)	HuSMP30 / Kd (μM)
Ca^2+^	7.89 ± 0.12	0.29 ± 0.13
Co^2+^	137.85 ± 49.09	6.66 ± 0.49
Mg^2+^	5.49 ± 2.9	2.73 ± 0.01
Zn^2+^	0.54 ± 0.02	0.88 ± 0.21

### Functional analysis of SMP30

The proteins purified from inclusion bodies were used to perform lactonase and OP hydrolase activity using γ-Thiobutyrolactone and Demeton-S as substrates respectively. The enzyme kinetics were performed to calculate the enzyme specificity (Km), maximum rate of reaction (Vmax), turn over number (Kcat) and overall catalytic efficiency (Kcat/Km) and summarized in tables (**Tables 2** and **3)**. Lactonase activity was observed in the presence of Ca^2+^ for both MoSMP30 and HuSMP30. In addition, MoSMP30 and HuSMP30 also showed lactonase activity in the presence of Co^2+^ and Zn^2+^ respectively, where Km values were also found to be lower suggesting higher specificity. The catalytic efficiency (Kcat/Km) of MoSMP30 were found to be six fold higher with Co^2+^ as compare to Ca^2+^, however, of HuSMP30, the Kcat/Km values were observed to be in the same order with Ca^2+^ as well as Zn^2+^ (**Table 2, S9 Fig**).

**Table 2:** Lactonase activity of MoSMP30 and HuSMP30 in the presence of different metal cofactors.

GTBL	MoSMP30				HuSMP30			
	Km (mM)	Vmax (mM min^-1^)	Kcat (min^-1^)	Kcat/ Km (mM^-1^ min^-1^)	Km (mM)	Vmax (mM min^-1^)	Kcat (min^-1^)	Kcat/ Km (mM^-1^ min^-1^)
Ca^2+^	39.49±7.9	2.86±0.9 ×10^−4^	3.88	0.098	19.8 ±2.9	4.53 ± 2.7×10^−4^	6.12	0.31
Co^2+^	19.17±7.88	8.57±3.5 ×10^−4^	11.65	0.60	ND	ND	ND	ND
Mg^2+^	ND	ND	ND	ND	ND	ND	ND	ND
Zn^2+^	ND	ND	ND	ND	9.68 ± 1.9	1.42±0.82×10^−4^	1.93	0.20

ND = Undetectable enzyme activity with 1mM metal concentration

**Table 3:** OP hydrolase activity of MoSMP30 and HuSMP30 in the presence of different metal cofactors.

Demeton-S	MoSMP30				HuSMP30			
	K_m_ (mM)	Vmax (mM min^-1^)	K_cat_ (min^-1^)	K_cat_/ K_m_ (mM^-1^ min^-1^)	K_m_ (mM)	Vmax mM Min^-1^)	K_cat_ (min^-1^)	K_cat_/ K_m_ (mM^-1^ min^-1^)
Ca^2+^	3.57 ± 1.5	0.085±0.028 ×10^−4^	0.116	0.032	17.45 ± 5	0.357±0.088 ×10^−4^	0.485	0.028
Co^2+^	ND	ND	ND	ND	ND	ND	ND	ND
Mg^2+^	ND	ND	ND	ND	ND	ND	ND	ND
Zn^2+^	4.69 ± 1.62	0.299±0.054 ×10^−4^	0.407	0.086	5.63 ± 1.51	0.41± 0.014×10^−4^	0.558	0.099

ND = Undetectable enzyme activity with 1mM metal concentration

On the other hand, OP hydrolase activity was observed for both the proteins with Ca^2+^ as well as Zn^2+^ (**Table 3**). However, with HuSMP30, the Km values were found to be lower in the presence of Zn^2+^ suggesting higher specificity. MoSMP30 showed less Km value with the Zn^2+^ and Ca^2+^, which suggested that in the presence of Zn^2+^ and Ca^2+^, MoSMP30 showed higher specificity with the OP substrate as compared to HuSMP30. However, there were no significant differences in the catalytic efficiencies of these proteins towards Demeton-S (**Table 3, S10 Fig**). There were no significant OP hydrolase activities detected with Co^2+^, though, lactonase activity was seen with Co^2+^ in case of MoSMP30 suggesting a metal-dependent functions.

## Discussion

Recombinantly expressing SMP30 proteins into the bacterial host often do not fold properly or expressed [16] which can be acheived by combination of low temperature for growth, co-expression of chaperones and osmolytes to increase the solubility of the protein [17]. Though the MoSMP30 and HuSMP30 both proteins were a cytoplasmic protein, it was aggregated more while expressing the protein in *E*. *coli* (BL21) [18]. This could be the result of the protein having hydrophobic residues at N- and C-terminal or due to more β-sheet conformations in the protein structure. Thus, in this study, both, MoSMP30 and HuSMP30 proteins were overexpressed, and purified from inclusion bodies in a large amount and more than 90% purity. The recovery of expressed proteins from inclusion bodies has a commercial advantage as a large amount of proteins can be achieved [16–20]. The proteins were able to show the lactonase and OP hydrolase activity recovered from inclusion bodies suggesting the proper refolding after solubilization. In this study, the CD spectroscopy data has shown that both the proteins in the presence of various metals acquired native conformations with slight variations in their secondary structures (**Fig 4**). This might be due to the plasticity of metal binding site, where non-specific metal ion binds and results in the generation of functional diversification of SMP30 proteins[21]. The affinity of the proteins with metals was assesed by UV-VIS spectroscopy using Hill fit equation [12]. The highest affinity of Zn^2+^ with MoSMP30 and Ca^2+^ with HuSMP30 were observed (**Table 1**).

Catalytic property of SMP30 has been studied and reported earlier [8, 22–24] which suggest the metal-dependent substrate specificities of the enzyme where the proteins were isolated from the liver of mouse, rat as well as recombinantly expressed and purified to perform the experiments [17,18]. However, in this study, the proteins were overexpressed in heterologous host and purified with greater yield. Previous study has demonstrated the Zn^2+^ dependent gluconolactonase activity of MoSMP30, and also its role in Ca^2+^ homeostasis[24]. In this study also, it was observed that Zn^2+^ was the preferred metal cofactor for the lactonase activity of HuSMP30 with GTBL substrate, whereas in case of MoSMP30, Co^2+^ was found to be the preferred metal cofactor for the same (**Table 2**). The promiscuous function, i.e. OP hydrolase acitivity was observed in the presence of both Ca^2+^ and Zn^2+^ as co-factors.

The mechanism of exhibiting promiscuity are different in different proteins, enzymes in particular. However, in this study co-factor based enzyme promiscuity has been demonstrated. The observations suggest that the metal-dependent promiscuous function of the SMP30 has evolved to acomplish new functions in nature[21,25]. This metal-dependent enzyme promiscuity is responsible for a regulatory mechanism which allows a single enzyme to specifically control different metabolic pathways and produce different metabolites [21,26,27]. This study also supports the importance of HuSMP30 in the calcium homeostasis as it has shown significantly high binding affinity (Kd = 0.29 ± 0.13 μM, **Table 1**) with Ca^2+^ [28–30].

## Conclusion

The current study provides sufficient evidence to express, purify and recover bioactive SMP30 protein from inclusion body using *E*. *coli* (BL21) host. The ability of inclusion body purification of this protein opens the floor to optimize proteins ability for hydrolysis of the toxic OP compounds Demeton-S in particular, which mimics V-types of nerve agents. HuSMP30 gene can be a very good starting point for the directed laboratory evolution of new enzyme for the OP hydrolysis. Furthermore, protein-engineering techniques can be applied to enhance the overall turnover of this enzyme for the OP degradation. Moreover, the biophysical characterization and metal-affinity with the divalent metals showing its adaptation towards maintaining the cellular calcium level.

## Supporting information

S1 Fig Cloning of MoSMP30 into Vector pET28 a (+). Agaroge gel images showing **(A**) the PCR amplified product of MoSMP30 gene, band size 879 bp, (**B**) confirmation of MoSMP30 gene cloning in pJET1.2 vector by restriction digestion, (**C**) preparation of vector by restriction digestion of the pET28 a (+) plasmid, and (**D**) confirmation of MoSMP30 gene cloning in pET28a vector by restriction digestion.
(JPG)

S2 Fig Cloning of HuSMP30 into pET28 a (+) vector. Agarose gel images showing **(A**) restriction digested HuSMP30 product with the restriction enzymes *Nde1* and *Xho1*, (**B**) vector preparation by the restriction digestion of the pET28 a (+) plasmid, band size ~5289 bp, and (**C)** confirmation of HuSMP30 gene cloning in pET28a vector by restriction digestion.
(TIF)

S3 Fig SDS-PAGE of MoSMP30 and HuSMP30 proteins from *E*. *coli* (BL21) strain after Coomassie brilliant blue (CBB) staining. HuSMP30 gene was inserted into pET28a vector and transformed into *E*. *coli* (BL21) cells. The protein concentrations were estimated by the using BCA method, and an equal amounts of proteins both from supernatants and pellets were resolved on 12% SDS-PAGE. Induced supernatant, induced pellet, un-induced supernatant, un-induced pellet, and standard protein marker were loaded in lanes 1, 2, 3, 4 and M respectively. The box indicates the induction of (**A**) MoSMP30 and (**B**) HuSMP30 proteins.
(TIF)

S4 Fig Purification of soluble proteins by Ni-NTA affinity chromatography. Purification of soluble fractions of proteins and the samples collected during several elution steps were analysed by 12% SDS-PAGE gel which shows (**A**) elutution fractions (E1, E2, E3, E4 and E5) collected for MoSMP30 and (**B**) elutution fractions (E1, E2 and E3) collected for HuSMP30.
(TIF)

S5 Fig Inclusion body (IB) preparation from the insoluble fractions. **12%** SDS-PAGE image showing (**A**) the samples collected during the subsequent purification steps 1, 2, 3, 4, 5 and 6 of IBs purification for MoSMP30. Lane 7 and 9 contain dissolved inclusion body protein and protein marker (ladder) respectively and (**B)** the samples collected during the subsequent purification steps 1, 2, 3, 4, 5 and 6 of IBs purification for HuSMP30. Protein marker (ladder) and dissolved inclusion body proteins were loaded in lane 7 and 9 respectively.
(TIF)

S6 Fig UV-VIS titration shown for HuSMP30 with Ca^2+^. For the calculation of metal binding affinity (Kd values) with different metals, UV-VIS data were acquired at 250-500nm wavelengths. Concentration dependent shift in the delta absorbance was observed at 333nm.
(TIF)

S7 Fig Calculation of Kd values for MoSMP30. Kd values were estimated for each of the metals by fitted to Hill equation with a non-linear curve at growth/Sigmoid model using Origin Pro software. The Y-axis represents the delta A_333nm_ and X-axis represents the different metal concentrations.
(TIF)

S8 Fig Calculation of Kd values for HuSMP30. Kd values were estimated for each of the metals by fitted to Hill equation with a non-linear curve at growth/Sigmoid model using Origin Pro software. The Y-axis represents the delta A_333nm_ and X-axis represents the different metal concentrations.
(TIF)

S9 Fig Enzyme kinetics by Lineweaver-Burk plots for the hydrolysis of GTBL. Double reciprocal plots for the calculation of kinetic parameters (Km and Vmax) were showing concentration dependent incraese in the hydrolysis of GTBL in the presence of (**A**) Ca^2+^ by MoSMP30, (**B**) Ca^2+^ by HoSMP30, (**C**) Co^2+^ by MoSMP30 and (**D**) Zn^2+^ by HuSMP30. Y-axis showing the reciprocal reaction velocity (OD/min), and X-axis showing the reciprocal substrate concentrations (mM). The error bar shows the standard error of the mean (SEM) calculated from triplicate experiments.
(TIF)

S10 Fig Enzyme kinetics by Lineweaver Burk Plots for the OP (Demeton-S) hydrolysis. Double reciprocal plots for the calculation of kinetic parameters (Km and Vmax) in the presence of Ca^2+^ and Zn^2+^ were able to show the activity. MoSMP30 showing increased rate of reaction with increasing conentration of the Demeton-S in the presence of (**A**) Ca^2+^ & (**C**) Zn^2+^. Similarily, HuSMP30 showing activity with (**B**) Ca^2+^ & (**D**) Zn^2+^. Y-axis showing the reciprocal reaction velocity (OD/min), and X-axis showing the reciprocal substrate concentrations (mM). The error bar shows the standard error of the mean (SEM) calculated from triplicate experiments.
(TIF)

S1 Table Proportion of secondary structure of MoSMP30 protein calculated by CDNN software using CD Spectrum OD (190-260nm). (DOCX)

S2 Table Proportion of secondary structure of HuSMP30 protein calculated by CDNN software using CD Spectrum OD (190-260nm). (DOCX)
