# Supplementary figures and images for: Comparative analysis of the metal-dependent structural and functional properties of mouse and human SMP30
Source: PLoS One. 2019 Jun 20;14(6):e0218629. doi: 10.1371/journal.pone.0218629 (PMC6586323; doi:10.1371/journal.pone.0218629)

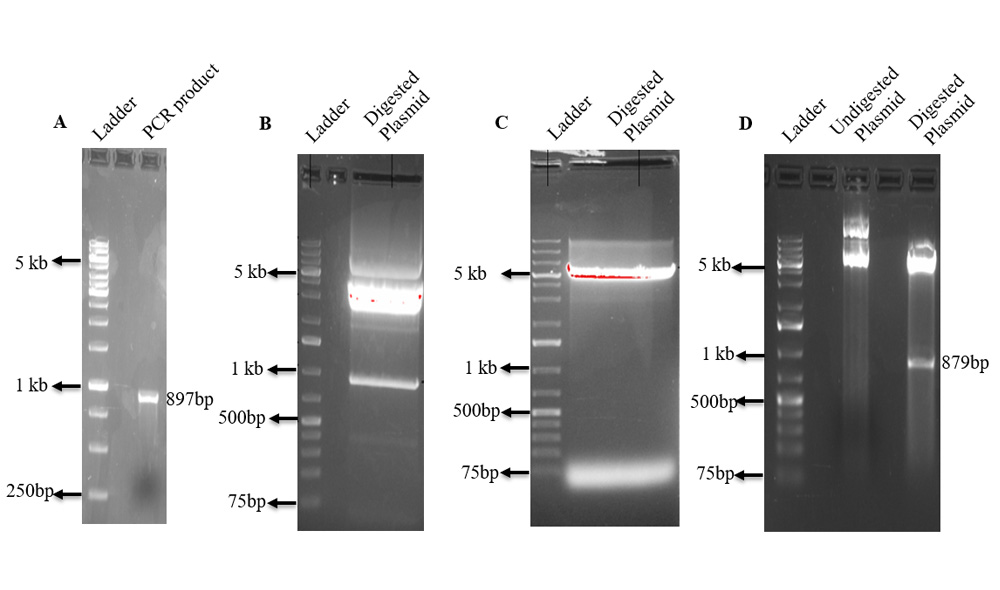

Supplement: S1 Fig — Agaroge gel images showing (A) the PCR amplified product of MoSMP30 gene, band size 879 bp, (B) confirmation of MoSMP30 gene cloning in pJET1.2 vector by restriction digestion, (C) preparation of vector by restriction digestion of the pET28 a (+) plasmid, and (D) confirmation of MoSMP30 gene cloning in pET28a vector by restriction digestion. (JPG) [file pone.0218629.s001.jpg]

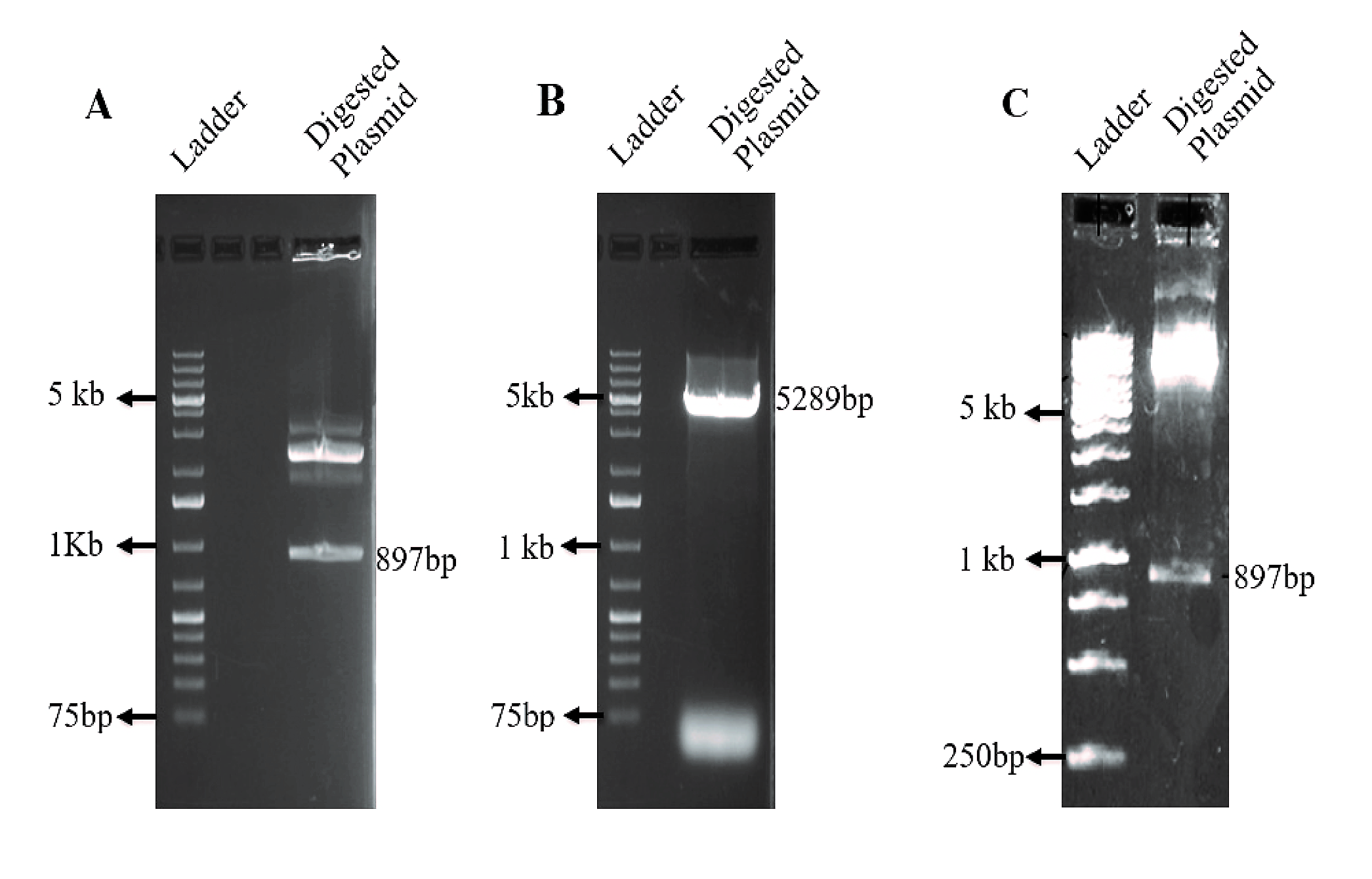

Supplement: S2 Fig — Agarose gel images showing (A) restriction digested HuSMP30 product with the restriction enzymes Nde1 and Xho1, (B) vector preparation by the restriction digestion of the pET28 a (+) plasmid, band size ~5289 bp, and (C) confirmation of HuSMP30 gene cloning in pET28a vector by restriction digestion. (TIF) [file pone.0218629.s002.tif]

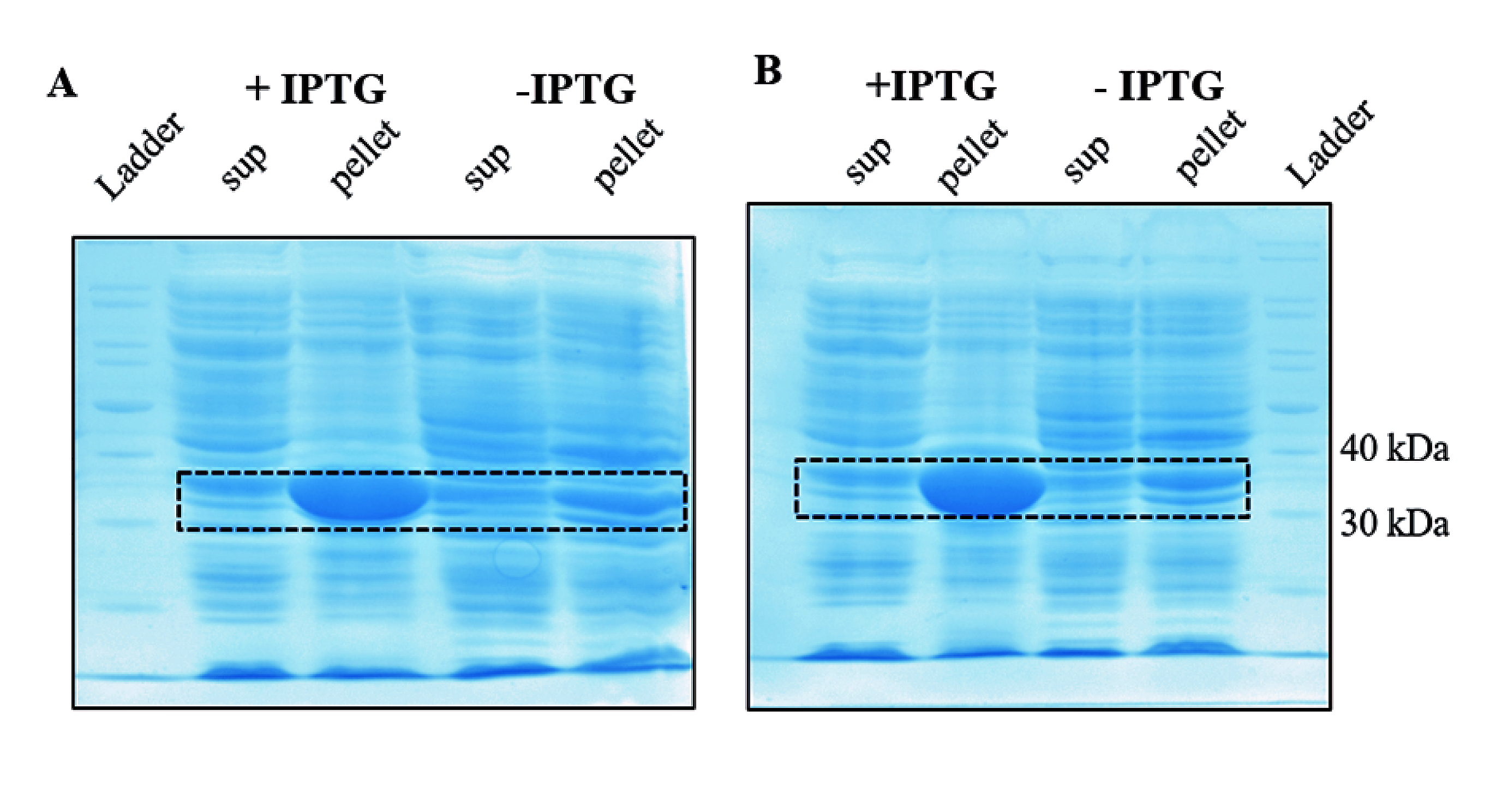

Supplement: S3 Fig — HuSMP30 gene was inserted into pET28a vector and transformed into E. coli (BL21) cells. The protein concentrations were estimated by the using BCA method, and an equal amounts of proteins both from supernatants and pellets were resolved on 12% SDS-PAGE. Induced supernatant, induced pellet, un-induced supernatant, un-induced pellet, and standard protein marker were loaded in lanes 1, 2, 3, 4 and M respectively. The box indicates the induction of (A) MoSMP30 and (B) HuSMP30 proteins. (TIF) [file pone.0218629.s003.tif]

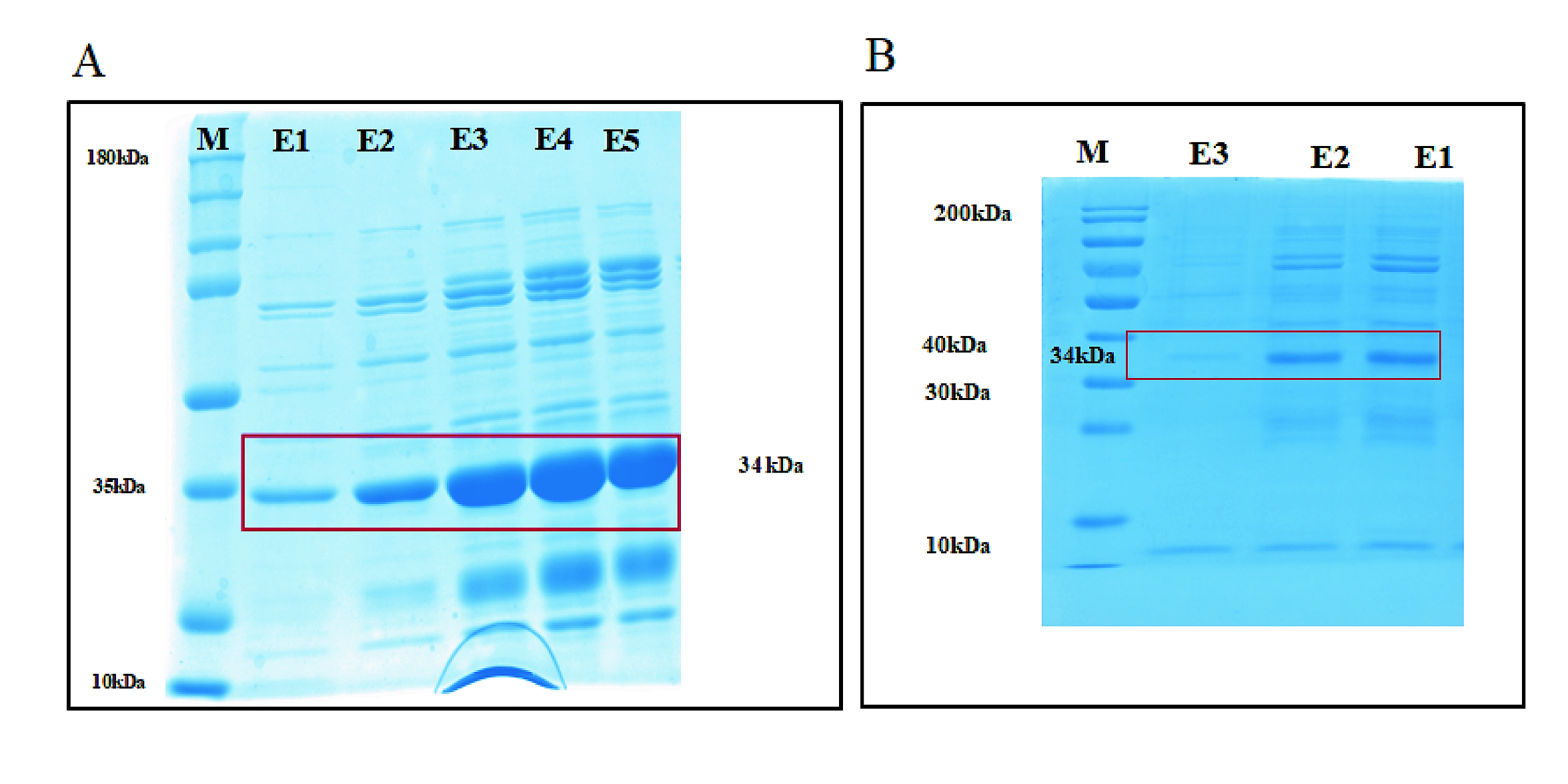

Supplement: S4 Fig — Purification of soluble fractions of proteins and the samples collected during several elution steps were analysed by 12% SDS-PAGE gel which shows (A) elutution fractions (E1, E2, E3, E4 and E5) collected for MoSMP30 and (B) elutution fractions (E1, E2 and E3) collected for HuSMP30. (TIF) [file pone.0218629.s004.tif]

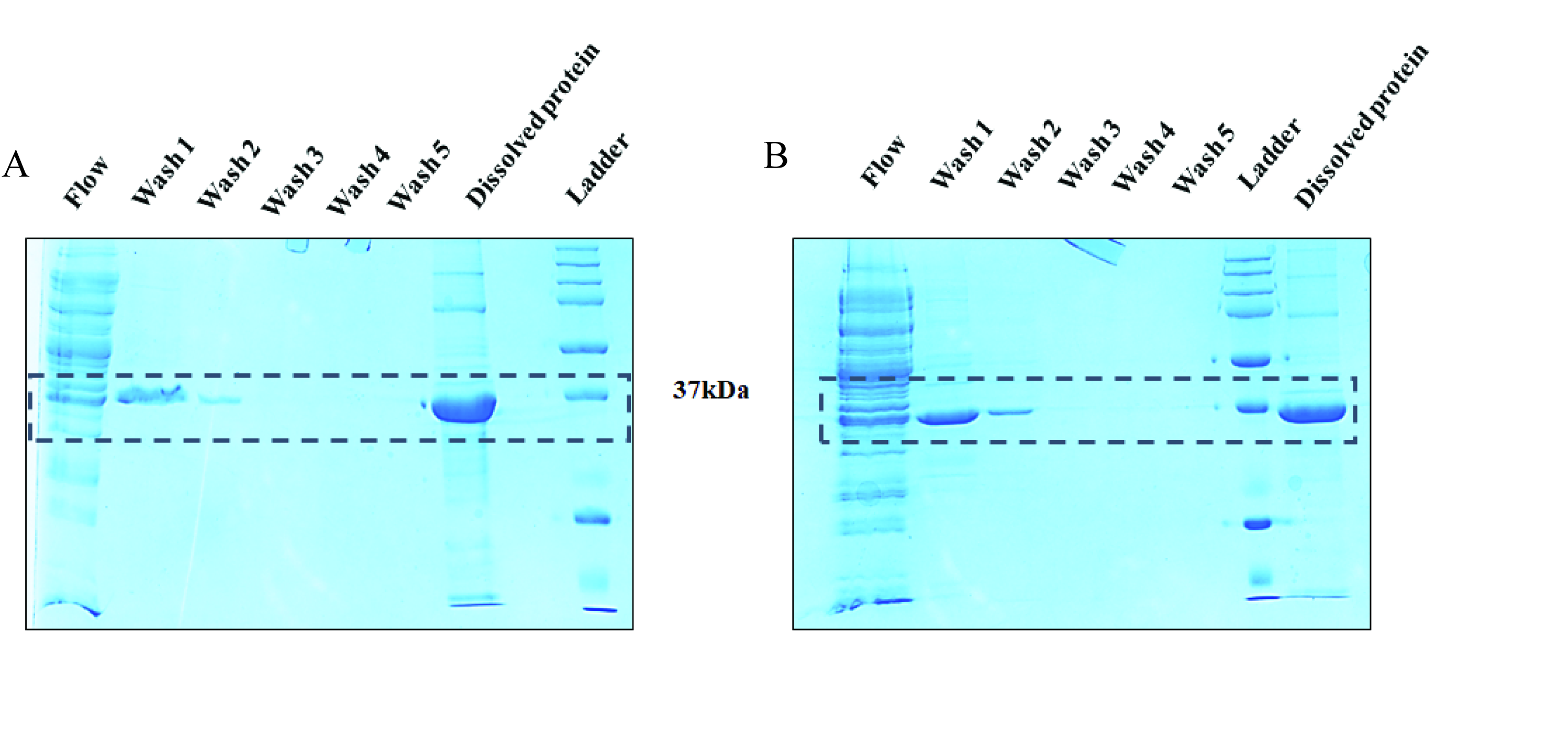

Supplement: S5 Fig — 12% SDS-PAGE image showing (A) the samples collected during the subsequent purification steps 1, 2, 3, 4, 5 and 6 of IBs purification for MoSMP30. Lane 7 and 9 contain dissolved inclusion body protein and protein marker (ladder) respectively and (B) the samples collected during the subsequent purification steps 1, 2, 3, 4, 5 and 6 of IBs purification for HuSMP30. Protein marker (ladder) and dissolved inclusion body proteins were loaded in lane 7 and 9 respectively. (TIF) [file pone.0218629.s005.tif]

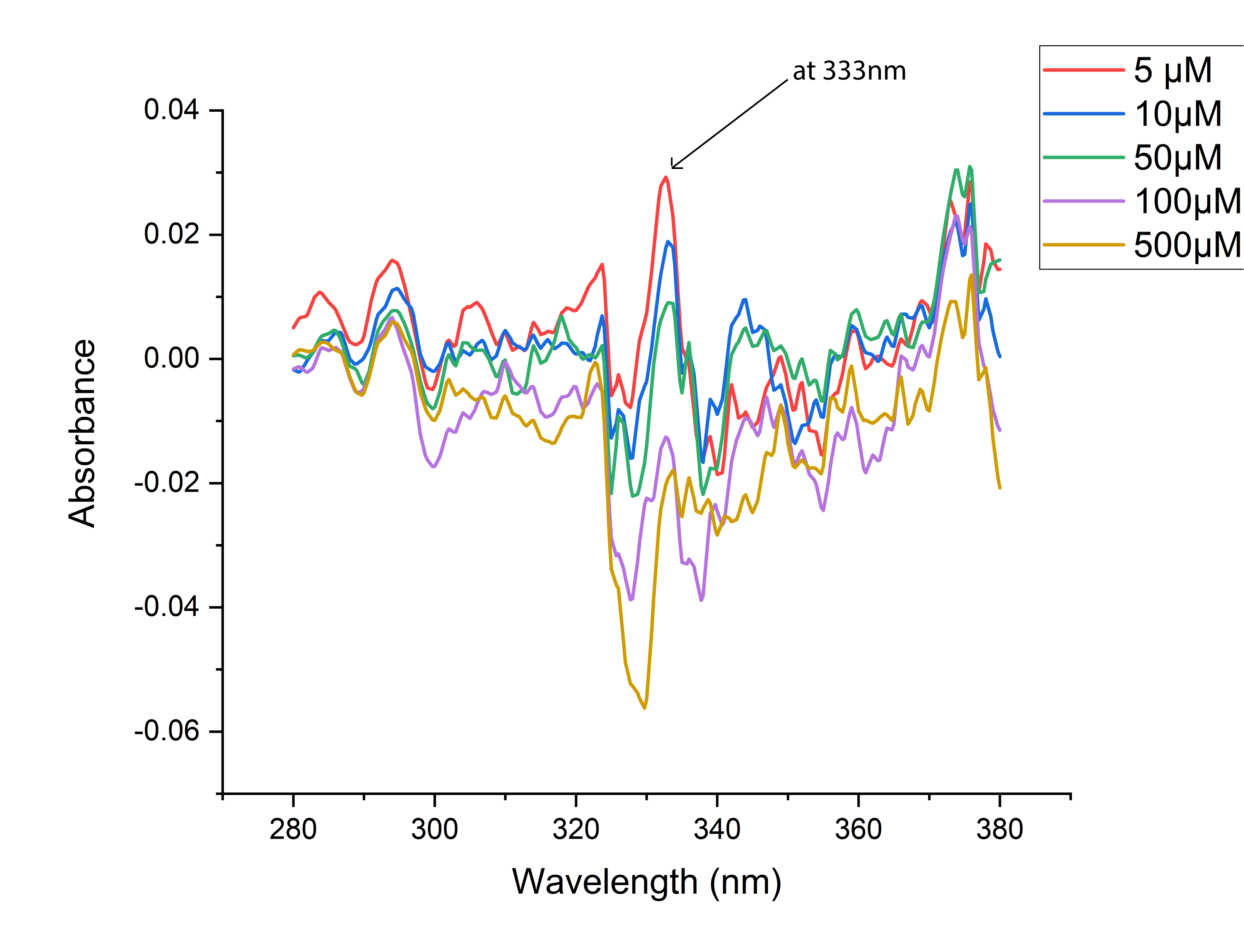

Supplement: S6 Fig — For the calculation of metal binding affinity (Kd values) with different metals, UV-VIS data were acquired at 250-500nm wavelengths. Concentration dependent shift in the delta absorbance was observed at 333nm. (TIF) [file pone.0218629.s006.tif]

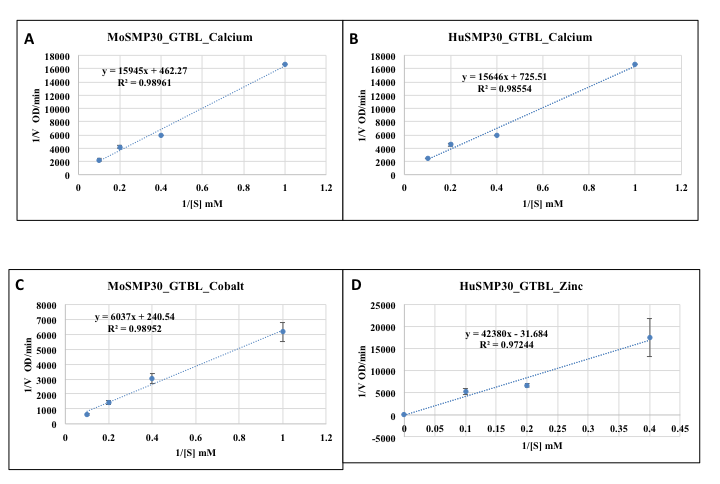

Supplement: S9 Fig — Double reciprocal plots for the calculation of kinetic parameters (Km and Vmax) were showing concentration dependent incraese in the hydrolysis of GTBL in the presence of (A) Ca2+ by MoSMP30, (B) Ca2+ by HoSMP30, (C) Co2+ by MoSMP30 and (D) Zn2+ by HuSMP30. Y-axis showing the reciprocal reaction velocity (OD/min), and X-axis showing the reciprocal substrate concentrations (mM). The error bar shows the standard error of the mean (SEM) calculated from triplicate experiments. (TIF) [file pone.0218629.s009.tif]

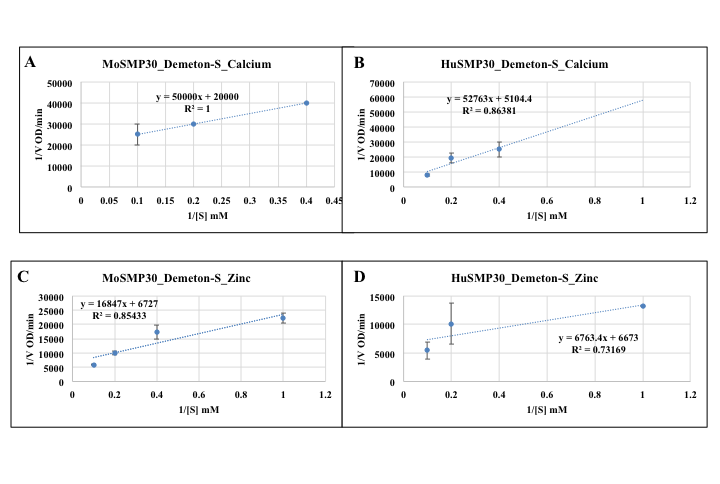

Supplement: S10 Fig — Double reciprocal plots for the calculation of kinetic parameters (Km and Vmax) in the presence of Ca2+ and Zn2+ were able to show the activity. MoSMP30 showing increased rate of reaction with increasing conentration of the Demeton-S in the presence of (A) Ca2+ & (C) Zn2+. Similarily, HuSMP30 showing activity with (B) Ca2+ & (D) Zn2+. Y-axis showing the reciprocal reaction velocity (OD/min), and X-axis showing the reciprocal substrate concentrations (mM). The error bar shows the standard error of the mean (SEM) calculated from triplicate experiments. (TIF) [file pone.0218629.s010.tif]
