# Supplementary material for: Comparative analysis of the metal-dependent structural and functional properties of mouse and human SMP30
Source: PLoS One. 2019 Jun 20;14(6):e0218629. doi: 10.1371/journal.pone.0218629 (PMC6586323; doi:10.1371/journal.pone.0218629)
Supplement: S1 Table — (DOCX) [file pone.0218629.s011.docx]

| **Mouse SMP30** | **Calcium Chloride** | | | **Cobalt Chloride** | | | **Magnesium Chloride** | | | | **Zinc Chloride** | | |
| --- | --- | --- | --- | --- | --- | --- | --- | --- | --- | --- | --- | --- | --- |
|  | **2mM** | **5mM** | **10mM** | **2mM** | **5mM** | **10mM** | **2mM** | **5mM** | **10mM** | **2mM** | | **5mM** | **10mM** |
| **Helix** | 36 | 33.5 | 31.2 | 32.4 | 40.1 | 39.5 | 33.7 | 32.6 | 35.6 | 38.9 | | 47.1 | 50.3 |
| **Anti-parallel** | 7.2 | 7.7 | 8.2 | 8 | 6.4 | 6.6 | 7.7 | 8 | 7.3 | 6.7 | | 5.4 | 5 |
| **Parallel** | 8.3 | 9 | 9.7 | 9.3 | 7.3 | 7.5 | 9 | 9.3 | 8.4 | 7.6 | | 6 | 5.4 |
| **Beta turn** | 16.1 | 16.5 | 16.9 | 16.7 | 15.5 | 15.6 | 16.5 | 16.7 | 16.2 | 15.6 | | 14.5 | 14.1 |
| **Random coil** | 31.9 | 33.6 | 35.4 | 34.4 | 29.3 | 29.6 | 33.5 | 34.2 | 32.1 | 30 | | 25.3 | 23.5 |

**S1 Table:** Proportion of secondary structure of MoSMP30 protein calculated by CDNN software using CD Spectrum OD (190-260nm). The average of OD was feeded in the CDNN software and the % given is plotted in the table above.
