# Supplementary material for: Comparative analysis of the metal-dependent structural and functional properties of mouse and human SMP30
Source: PLoS One. 2019 Jun 20;14(6):e0218629. doi: 10.1371/journal.pone.0218629 (PMC6586323; doi:10.1371/journal.pone.0218629)
Supplement: S2 Table — (DOCX) [file pone.0218629.s012.docx]

| **Human SMP30** | **Calcium Chloride** | | | **Cobalt Chloride** | | | **Magnesium Chloride** | | | | **Zinc Chloride** | | |
| --- | --- | --- | --- | --- | --- | --- | --- | --- | --- | --- | --- | --- | --- |
|  | **2mM** | **5mM** | **10mM** | **2mM** | **5mM** | **10mM** | **2mM** | **5mM** | **10mM** | **2mM** | | **5mM** | **10mM** |
| **Helix** | 38.6 | 44.7 | 69 | 43.1 | 41.8 | 46 | 36.4 | 43.5 | 37.5 | 36.9 | | 37.7 | 40.6 |
| **Anti-parallel** | 6.7 | 5.7 | 3 | 6 | 6.2 | 5.6 | 7.1 | 5.9 | 6.9 | 7.1 | | 6.9 | 6.4 |
| **Parallel** | 7.7 | 6.4 | 3 | 6.7 | 7 | 6.2 | 8.2 | 6.7 | 7.9 | 8.1 | | 7.9 | 7.2 |
| **Beta turn** | 15.6 | 14.8 | 11.8 | 15 | 15.3 | 14.7 | 16 | 15 | 15.8 | 16 | | 15.8 | 15.4 |
| **Random coil** | 30.2 | 26.6 | 14.9 | 27.5 | 28.1 | 25.7 | 31.7 | 27.3 | 30.9 | 31.2 | | 30.7 | 28.9 |

**S2 Table:** Proportion of secondary structure of HuSMP30 protein calculated by CDNN software using CD Spectrum OD (190-260nm). The average of OD was feeded in the CDNN software and the % given is plotted in the table above.
